# Supplementary material for: Early Pathogenesis of Wesselsbron Disease in Pregnant Ewes
Source: Pathogens. 2020 May 13;9(5):373. doi: 10.3390/pathogens9050373 (PMC7281025; doi:10.3390/pathogens9050373)
Supplement: Supplementary file 1 [file pathogens-09-00373-s001.pdf]

Supplementary Materials: www.mdpi.com/xxx/s1, Figure S1: WSLV infection in human brain cell lines.

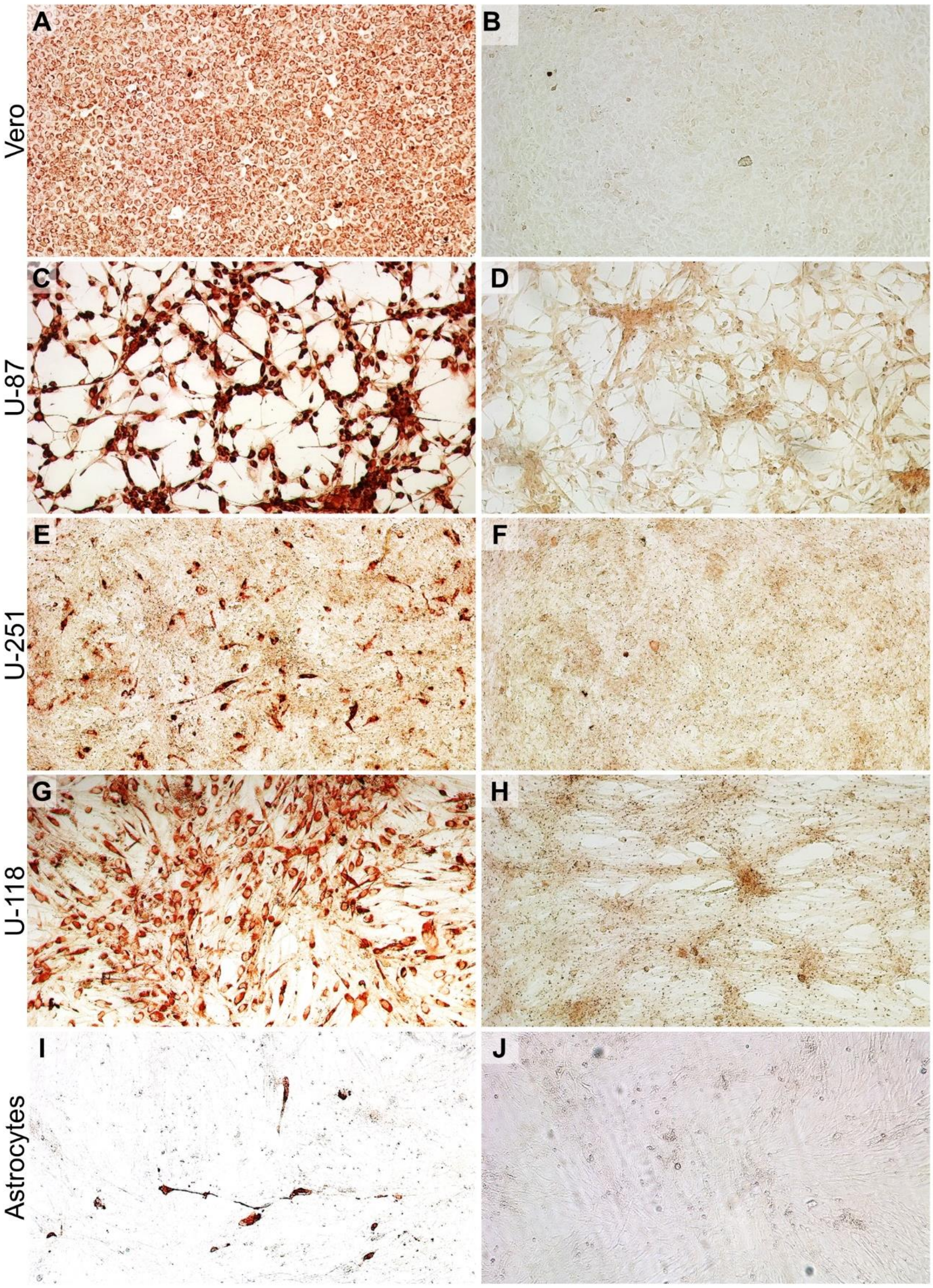

Figure S1. WSLV and mock infection of Vero cells (**A**,**B**), human glioma cells (U-87, **C** and **D**; U251, **E** and **F**; U118, **G** and **H**) and human astrocytes (**I** and **J**). All cells were infected with a MOI of 0.1(based on WSLV titre determined on Vero cells) and incubated for 3 days. An immunoperoxidase monolayer assay (IPMA) was subsequently performed to visualise infected cells. Briefly, cells were fixed and permeabilised at 3 dpi with 4% paraformaldehyde (10 min) and ice-cold methanol (10 min). After permeabilisation, the plates were incubated with rabbit polyclonal antibodies recognizing WSLV NS1, followed by incubation with polyclonal rabbit-α-mouse immunoglobin/HRP antibody (Dako, Denmark) as secondary antibody and 3-Amino-9-ethylcarbazole (AEC; Sigma-Aldrich) as substrate.
